# Effects of interactions in natural gas/water/rock system on hydrocarbon migration and accumulation

**DOI:** 10.1038/s41598-021-01653-0

**Published:** 2021-11-11

**Authors:** Lin Jiang, Wen Zhao, Jianguo Huang, Yang Fan, Jiaqing Hao

**Affiliations:** 1 Research Institute of Petroleum Exploration and Development, PetroChina, Beijing, 100083 People’s Republic of China; 2 State Key Laboratory of Petroleum Resources and Prospecting, China University of Petroleum (Beijing), Beijing, 102249 People’s Republic of China; 3 Northeast Petroleum University, Daqing, 163318 People’s Republic of China

**Keywords:** Fossil fuels, Petrol

## Abstract

The study of natural gas accumulation process in tight formation has become the focus of the petroleum industry. One of the priorities is the effects of interactions in natural gas/water/rock system on hydrocarbon migration and accumulation process. On the macroscopic scale, we investigate the interactions in natural gas/water/rock system by formation fluorescence test and production data analysis. One the microscopic scale, the mechanisms are revealed by mathematical analysis and experimental methods considering the variation of geological temperature and pressure. The effects of interactions in natural gas/water/rock system are also simulated by numerical simulation. The results are visualized and quantified. A novel semi-analytical method based on a physical experiment is proposed to calculate the temperature- and pressure-dependent contact angle and interface tension which reflect the interactions in the natural gas–water–rock system. This semi-analytical is embedded in the numerical simulation during the simulation of the natural gas charging process. The results indicate that with the increase of geological temperature and pressure, the contact angle will increase and the interface tension between natural gas and water will decrease. The capillary resistance in the formation will be reduced. Since the decrease of capillary resistance, the natural gas can be charged into smaller pores, so that the actual charging threshold is lower than the one originally obtained under present reservoir conditions. After considering the temperature and pressure during the accumulation process, some sand bodies that were thought not to be charged may have natural gas accumulate.

## Introduction

**Figure 1:**
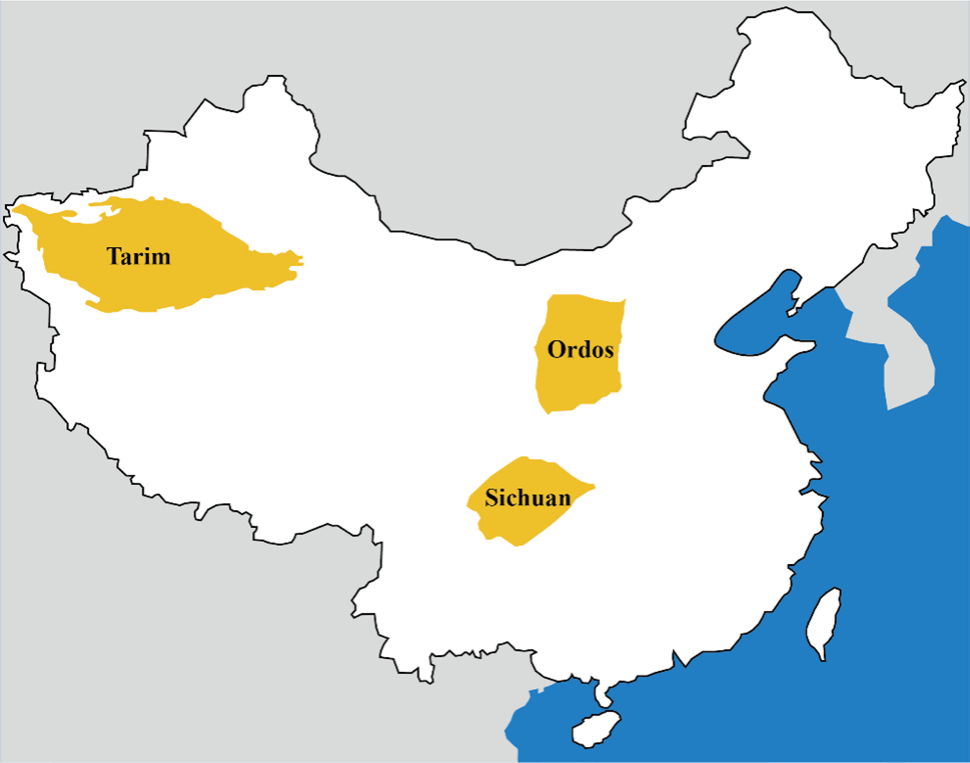
Three main tight gas distribution basins, China.

As a high-quality fuel and clean fossil energy, natural gas is playing an increasingly important role in the adjustment of China's energy structure^1^. In recent years, we have witnessed a skyrocketing increase of tight gas in China. In 2020, tight gas production accounts for more than 19% of the natural gas production in China^2^. As shown in Fig. 1, there are three main tight gas distribution basins in China, Ordos Basin, Tarim Bain, and Sichuan Basin. Numbers of tight gas-bearing formation have been found in these basins, such as Shanxi Formation (Sulige gas field and Daniudi gas field) in Ordos Basin^3^, Triassic Xujiahe Formation (Guangan gas field and Anyue gas field in Sichuan Basin^4^. Guangan and Anyue gas fields have been successfully developed, Hechuan gas field is in urgent need of development. It is of vital significance to study the hydrocarbon accumulation process for tight gas exploration and development^5^. A large number of exploration practices have confirmed that tight sandstone reservoirs have distinct characteristics of formation and distribution, and it is hard for conventional petroleum geology theories and methods to be directly converted to tight gas accumulation^6,7^.

Hydrocarbon accumulation is the process that the hydrocarbon generated by the source rock migrates to the reservoir through the hydrocarbon carrier system, which determines the scale and distribution of the reservoirs and is very important to hydrocarbon exploration and development^8^. Since the tight formation has the characteristics of the wide distribution of micro- and nano-pores, unsatisfied physical properties, larger specific surface area, and strong heterogeneity of mineral distribution, the effects of interactions of the natural gas/water/rock system in tight formation cannot be ignored during the hydrocarbon migration and accumulation process^9,10^. Different from conventional resources, buoyancy is no longer the main driving force during the hydrocarbon migration process^11^. The expansion force of hydrocarbon generation becomes the main force in tight formation^6,7^ When the expansion force of hydrocarbon generation is greater than the capillary force of the formation, the hydrocarbon will migrate and accumulate in the reservoir. As the main resistance during the hydrocarbon migration process, the capillary force determines whether hydrocarbon can be charged into the reservoir^11^. In this situation, the interactions of the natural gas/water/rock system are of vital significance for the estimate of capillary force. Three factors have an important influence on the capillary force, including interfacial tension of the two-phase fluid, capillary radius, and wettability of reservoir rock^12,13^. Pore radius is an inherent property of the reservoir. The interface tension, and wettability are both temperature- and pressure-dependent properties. Typically, it is assumed that the temperature may significantly affect the wettability performance on rock surfaces and the interfacial tension of two-phase fluid, the pressure dependence of the wettability and interface tension is relatively weak^14,15^. Many researchers investigate the impact of temperature and pressure on wettability and interface tension. However, few studies evaluate them under real geological conditions. In this study, we will investigate the impact of temperature and pressure on the wettability and interface tension under geological conditions, give a convenient calculation method of these temperature- and pressure-dependent parameters. The analytical algorithm will be embedded into numerical simulation software to simulate the hydrocarbon migration and accumulation process. The research on wettability in tight reservoirs mainly involves the measurement of wettability^16^, the influencing factors of wettability^17^, the influence of wettability on fluid flow^14^, and the modification of wettability^18^. However, the research on the wettability of tight reservoirs mainly focuses on reservoir evaluation^19^, oil and gas field development^9^, and improving oil and gas recovery^20^, etc. A profound understanding of the effects of wettability is still scarce for hydrocarbon accumulation in a tight formation, giving challenges to the accurate prediction of tight gas potential areas and development. In this study, we will consider the effects of wettability on hydrocarbon charging and accumulation in a tight sandstone formation.

Accurate determination of hydrocarbon accumulation period is of great significance for studying hydrocarbon migration and accumulation process^21^. Traditional analysis methods determine the hydrocarbon accumulation period indirectly, such as studying the tectonic development history^22^, the trap formation history^23^, and the hydrocarbon generation and expulsion history of source rocks^24^. In recent years, great progress has been made in the study of hydrocarbon accumulation stages. Some new analytical techniques and methods, such as fluid inclusion analysis^25^, autogenic mineral isotopic dating^26^, hydrocarbon-generation kinetics^27^, and hydrocarbon geochemistry^28^, have provided more accurate analytical means for determining hydrocarbon accumulation stages. Fluid inclusions are the parts of materials that are encapsulated in the crystal lattice defects or holes of the diagenetic and ore-forming fluids and during the process of mineral crystallization and growth. Due to its widespread existence and containing rich information of accumulation and mineralization. During the formation process, it retains important information such as temperature, pressure, composition of underground fluids, which provides geochemical evidence for the study of hydrocarbon accumulation and paleo-fluid composition^29^. In this study, we investigate the microscopic characteristics, types, and homogenization temperature distribution of fluid inclusions in the Triassic Xujiahe Formation of the Hechuan gas field systematically, and study the gas accumulation stage of the Hechuan gas field. To the best of our knowledge, this is the first study on natural gas migration and accumulation considering the interaction in natural gas/water/rock system by physical and numerical simulation method.

The paper structure is organized as follows. In “Wettability characteristics in the formation”, through the fluorescence analysis, we found that oil-wet reservoirs are conducive to the charging of natural gas and the production of natural gas. In “Interactions in natural gas–water–rock system”, we use contact angle to reflect the interactions between rock and fluids and interface tension to reflect the interactions between fluids. A novel semi-analytical method is present to calculate the interface tension which is geological temperature- and pressure-dependent between fluids. This method is embedded in the numerical simulation method in “Numerical simulation set up”. Our work investigates the interactions in the natural gas/water/rock system, sheds light on natural gas migration and accumulation in Xujiahe Formation, Sichuan Basin, China.

## Wettability characteristics in the formation

**Figure 2:**
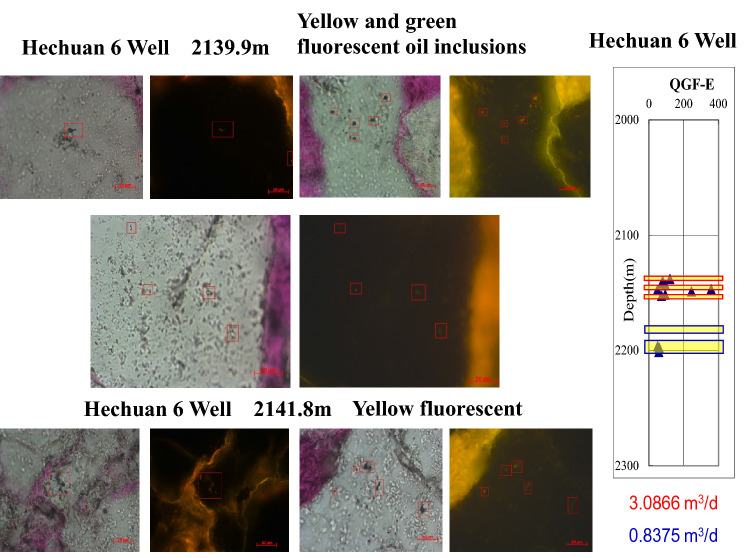
Micrographs of fluid inclusions of Xujiahe Formation in the Hechuan area of Hechuan 6 well.

According to the analysis of fluid inclusions thin sections in Hechuan area, it is found that oil inclusions are widely developed in high-production layers. There are mainly yellow and green fluorescent oil inclusions, indicating that there was a process of crude oil charging (Fig. 2).

**Figure 3:**
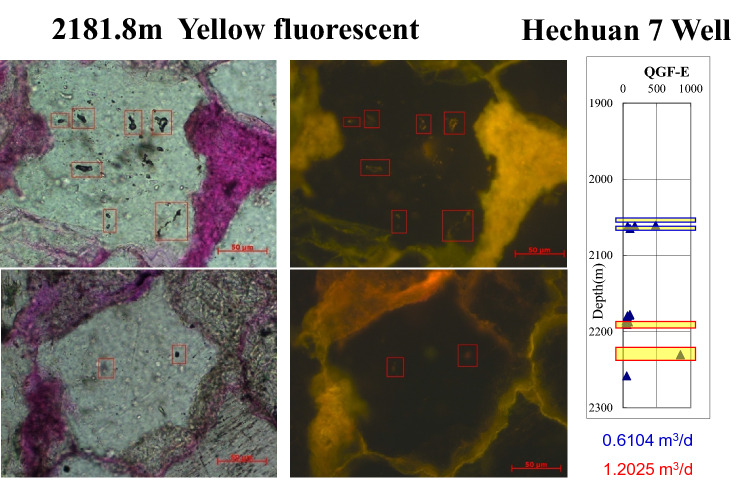
Micrographs of fluid inclusions of Xujiahe Formation in the Hechuan area of Hechuan 7 well.

According to the analysis of inclutions, the existence of oil inclusions is a sign of oil migration in the reservoir (Fig. 3), and the oil migration process will affect the wettability of the reservoir, usually changing the wettability of the reservoir gradually from hydrophilic to hydrophobic (oil-wet).

The fluorescence spectroscopy method can quickly and qualitatively evaluate the wettability of formation^30^. Aromatic hydrocarbons and polar compounds in hydrocarbon spontaneously fluoresce when excited by ultraviolet light^31^. QGF-E spectrum represents the fluorescence characteristics of hydrocarbon adsorption on the surface of reservoir particles, which can be used to determine the present or residual oil reservoirs in exploration and drilling evaluation.

In this work, we adopted QGF-E technique to analyze the fluorescence characteristics of reservoir. The specific operation steps are as follows:

1. Clean the surface of reservoir particles with deionized water;
2. After drying, use dichloromethane to extract, the quantitative fluorescence analysis results of the extracted liquid can represent the situation of free hydrocarbon;
3. Treat the surface of particles with hydrogen peroxide and dilute hydrochloric acid and wash them with deionized water;
4. After drying, use dichloromethane to extract;
5. The results of quantitative fluorescence analysis of the extraction solution can represent the adsorption of hydrocarbon.

**Figure 4:**
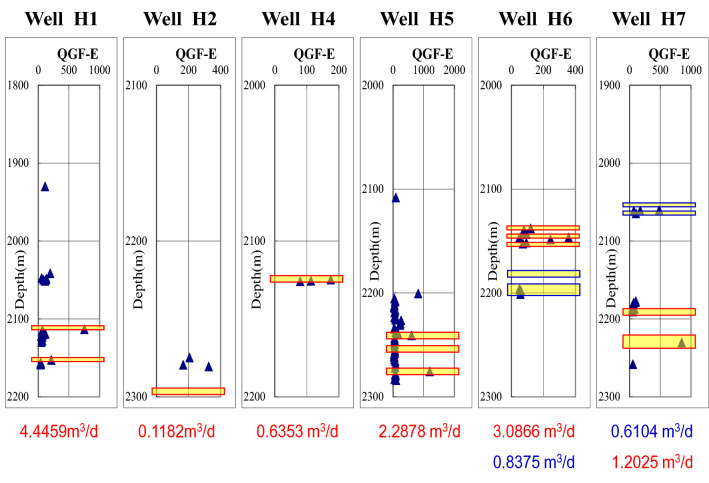
Comparison diagram of quantitative fluorescence analysis of the first extract of gas test layer in Hechuan area. The blue triangle represents the value of QGF-E value. The width of the yellow bar represents the natural gas production. The wider the yellow bar is, the larger the natural gas production is. Where the reservoir fluorescence is strong, the production is also high.

**Figure 5:**
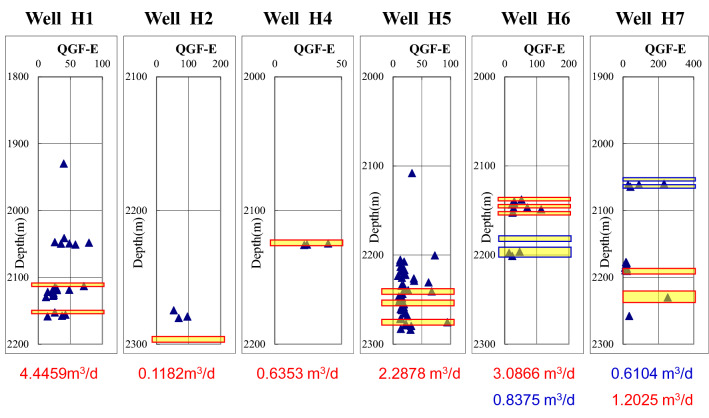
Comparison diagram of quantitative fluorescence analysis of the second extract of gas test layer in Hechuan area. The blue triangle represents the value of QGF-E value. The width of the yellow bar represents the natural gas production. The wider the yellow bar is, the larger the natural gas production is. Where the reservoir fluorescence is strong, the production is also high.

The existence and content of adsorbed hydrocarbon and free hydrocarbon can reflect the oil-wet degree of reservoir. Figures 4 and 5 show the results of the first and the second quantitative fluorescence analysis and the second quantitative fluorescence analysis of the extracted liquid in Hechuan area.

Quantitative fluorescence analysis of the first extraction solution and the quantitative fluorescence analysis of the second extraction solution in Hechuan area both show that the fluorescence intensity of the particles in the high-producing wells and the particles in the high-producing layers is usually strong.

Oil inclusions are widely developed in high-yielding reservoirs in Hechuan area, which indicates that there was a process of oil charging in high-yielding reservoirs. The results of quantitative fluorescence analysis of the two extracts were consistent, indicating that the fluorescence intensity of the high-producing Wells was usually higher, indicating that there are still adsorbed hydrocarbon and free hydrocarbon on the surface of the high-producing reservoirs. Therefore, wettability is an important factor controlling the gas bearing ability of tight reservoirs. Oil-wet reservoirs are conducive to the charging of natural gas and the production of natural gas.

The characteristics of reservoir wettability are macroscopic expressions of interactions in natural gas/water/rock system at the micro level. In next sections, we will analyze the interactions in natural gas–water–rock system, and reveal the mechanisms of the effects of wettability.

## Interactions in natural gas–water–rock system

The wettability in natural gas–water–rock system reflect the interactions of fluid–rock and fluid–fluid in the natural gas–water–rock system. In this section, we will propose a novel semi-analytical calculation method of contact angle and interface tension. This method is convenient to be used in the geological engineering field and will be embedded in the numerical simulation software for the simulation of hydrocarbon migration and accumulation.

### Contact angle

The wettability shows the interactions between rock walls and fluids, this character can be expressed by the contact angle. The direct integration of the augmented Young–Laplace equation of capillarity can be given as:

1 $${P}_{c}=\frac{\sigma }{r}+\Pi \left({f}^{*}\right),$$

where $\Pi$ is the disjoining pressure, σ is the interface tension, r is the interface curvature.

The relationship between the disjoining pressure and contact angle is^32^

2 $$cos\theta =1+\frac{{f}^{*}\Pi \left({f}^{*}\right)+\underset{{f}^{*}}{\overset{\infty }{\int }}\Pi (f)df}{\sigma }.$$

In DLVO theory, the interaction forces between the solid wall and gas–water interface are either repulsive or attractive. Three forces constitute the disjoining pressure: the electrostatic force (*F*_*e*_), the structural force (*F*_*s*_), and the van der Waals force (*F*_*vdW*_).

In tight sandstone, the contribution of electrostatic force and structural force is limited and can be neglected. The van der Waals forces depend on the distance between gas/water surface, for think film interactions, and can be given by^33^

3 $${F}_{vdW}=\frac{-{H}_{vdW}}{6\pi {f}^{3}}$$

where *f* is the film thickness, *H*_vdW_ is the Hamaker constant for the interactions between gas/water system and can be calculated as:

4 $${H}_{vdW}=(\sqrt{{H}_{ww}}-\sqrt{{H}_{gg}})(\sqrt{{H}_{ww}}-\sqrt{{H}_{ss}}),$$

where *H*_ww_ is the is Hamaker constant for water/water, *H*_gg_ is Hamaker constant for gas/gas, and *H*_ss_ is Hamaker constant for solid/solid. These Hamaker constants are calculated in terms of the refractive index and the dielectric permittivity of the interface^34^.

The contact angle can be calculated with Eq. (5):

5 $$cos\theta =1-\frac{{H}_{vdW}}{24\pi {{\sigma f}^{*}}^{2}}.$$

**Figure 6:**
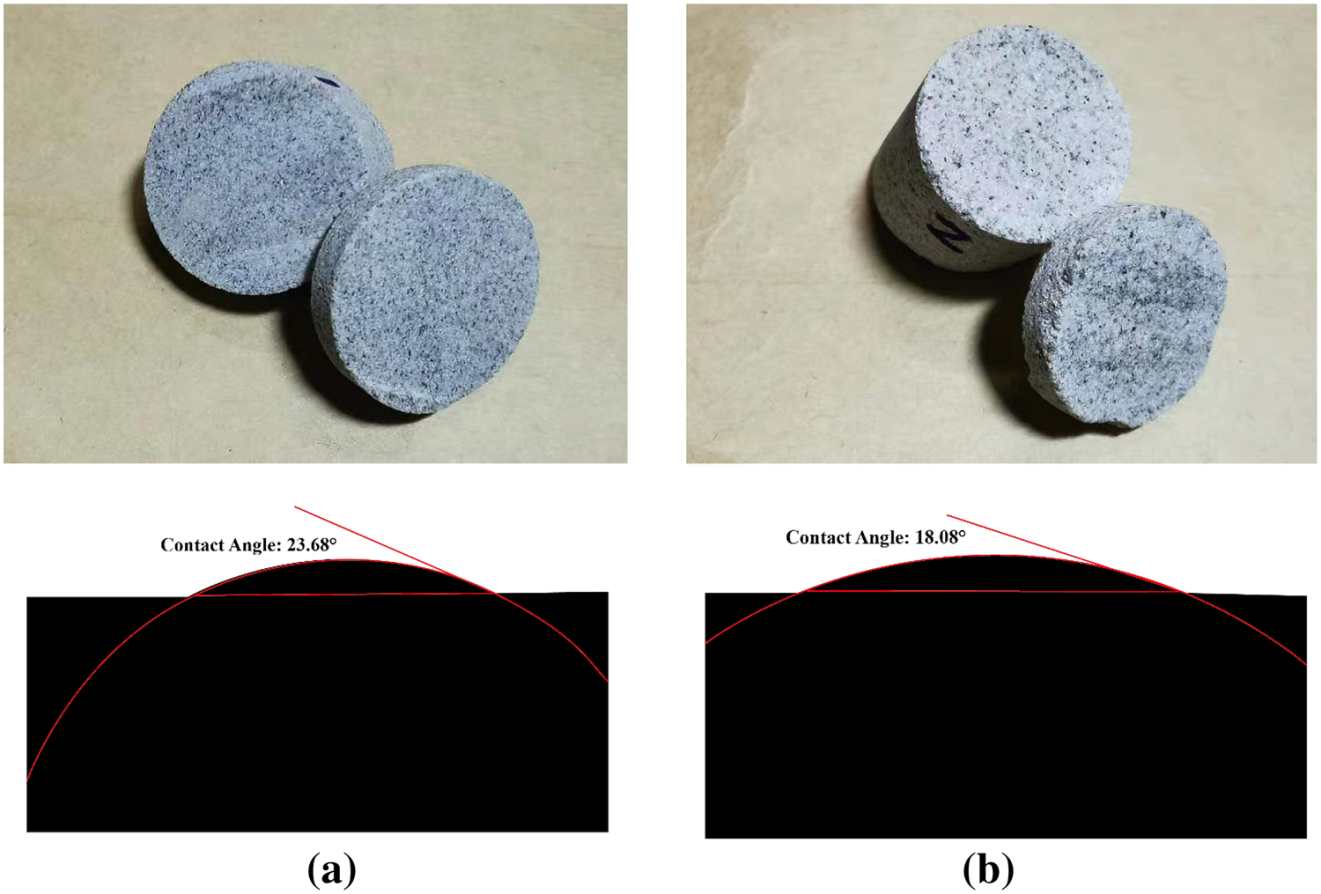
The snapshots of the captive droplet method. The black is the water and rock surface, the red line is the datum line used for the determination of contact angle.

The Hamaker constant for the interactions between gas/water systems varies depending on the different formations. To make our calculation method closer to the actual formation condition. We determine the value of the Hamaker constant used in this work by measuring the contact angle of the real core from the Hechuan area, the Xujiahe Formation. The captive droplet method is adopted to test the contact angle of five samples from Xujiahe Formation. Figure 6 shows two snapshots of the captive droplet test. The contact angle ranges from 18.08° to 23.68°, the average is 20.3°.

We determined the contact angle according to the national petroleum testing standard (Test method of reservoir rock wettability, SY/T 5153-2017). The contour image analysis method is adopted in our research. The main steps are as follows:

1. Place the rock sheet on a support frame;
2. Measure the temperature and pressure conditions;
3. Drop a drop of water on the rock sheet (as shown in Fig. 6);
4. Take a snapshot of water drop, and measure the contact angle by angular measure software.

**Figure 7:**
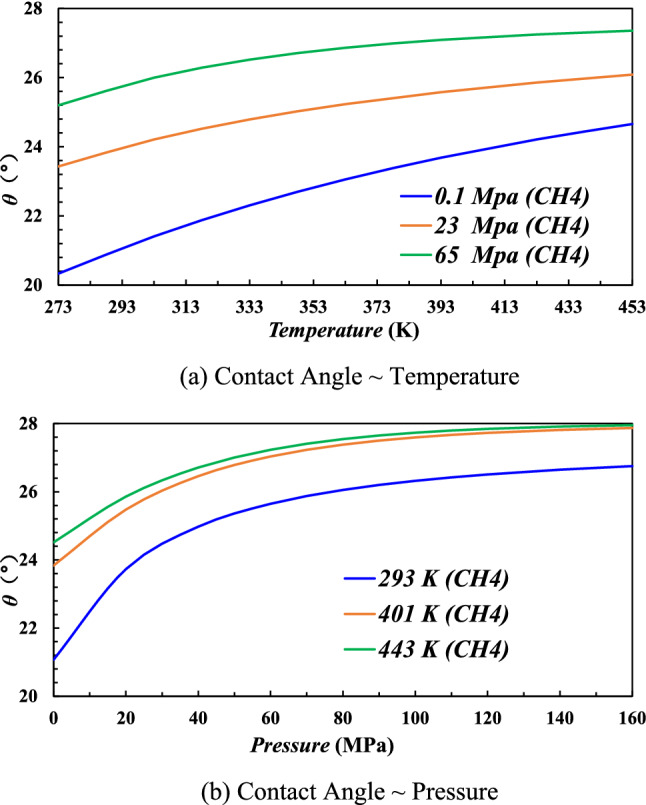
Contact angle under different pressure and temperature.

Figure 7 shows that the contact angle displays an increasing trend with the increase pressure and temperature. As shown in Fig. 7a, under the present reservoir pressure (P = 23 MPa), when temperature increases from 333 to 443 K, the contact angle increases from 24.7° to 26.08°, increasing about 5.59%. In Fig. 7b, under the present reservoir temperature (T = 333 K), when pressure increases from 23 to 65 MPa, the contact angle increases from 25.41° to 27.22°, increasing about 7.12%. Note that the contact angle increases with the increase of pressure and temperature, which indicates that the formation is less water-wet (or more gas wet) when gas is charged into the formation.

### Interface tension (IFT)

As a significant parameter to describe the interactions between gas and water, IFT is one of the quantitative indexes of molecular behavior at the natural gas/water interface. From the micro perspective, IFT can reflect the difference in intermolecular interactions, chemical nature, and molecular densities of gas and water which are strongly dependent on the variation of temperature and pressure.

In previous research, large amounts of experiments, theoretical methods, and molecular dynamic (MD) simulations have been adopted to characterize the influences of pressure and temperature on interface tension of pure or mixed hydrocarbon systems. In this work, a simple semi-empirical model (Eq. 6) correlating the interface tension of gas/water with density difference and reduced temperature is adopted^35,36^. This model is simple and effective enough to be used in engineering applications and numerical calculations, and it can be easily incorporated into numerical simulators.

6 $${\sigma }_{gw}=A{({\rho }_{w}-{\rho }_{g})}^{B}{T}_{r}^{C}+D.$$

**Figure 8:**
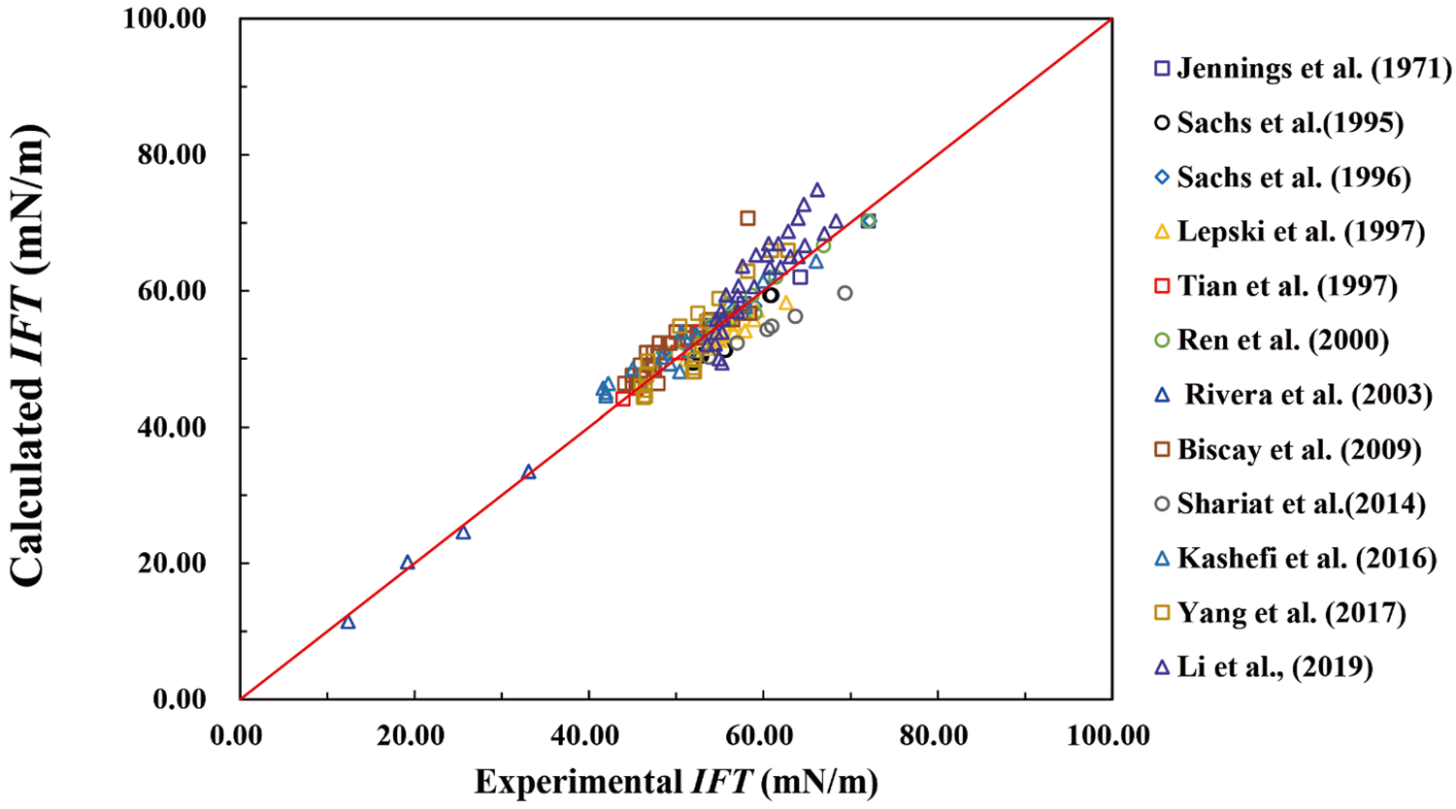
Comparison of the IFT from published experiments and simulations with the calculated results by the proposed model. The diagonal line is the guided line for convenient comparison of our semi-empirical model and 167 published cases.

In this semi-empirical model (Eq. 6), A, B, C, D are four constants that depend on the gas properties. In this work, we restricted our research on natural gas, 167 methane/water cases collected from published work are used to get the optimal constants with a nonlinear regression routine. The temperature in the 167 cases ranges from 273 to 373 K, and the pressure range from 0.1 MPa to 100 MPa. As shown in Fig. 8, our semi-empirical model fits well with the 167 cases with the parameters A = 82.61, B = 6.64, C = − 2.25, D = 41.85. The errors of the majority of these cases are less than 5%, and the details are in Supplementary Appendix A.

**Figure 9:**
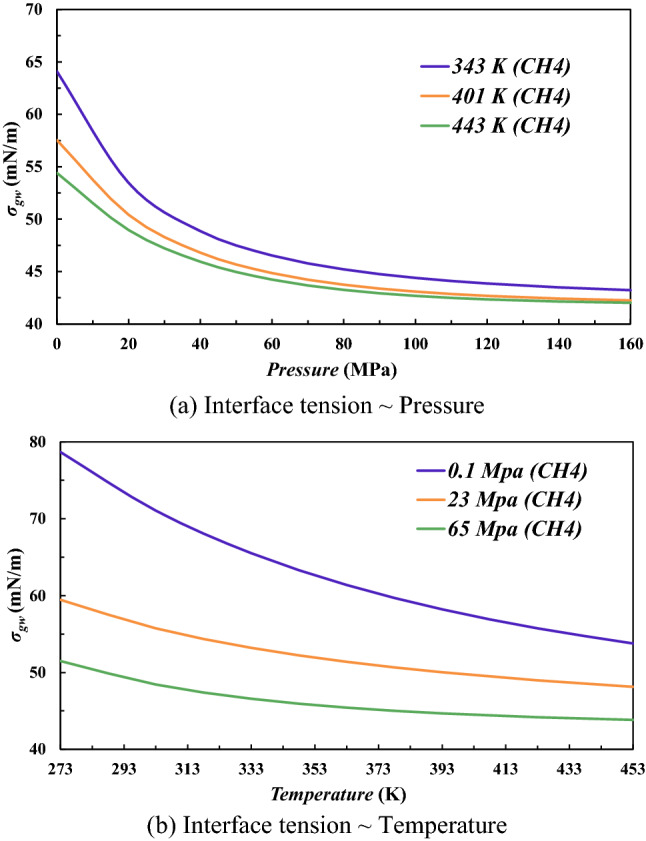
IFT of the CH4 and water system under different pressure and temperature. Different color indicates the variation of interface tension under different temperature or pressure.

Figure 9a shows the variation of CH_4_/water interface tension under different pressure. Under the condition of identical temperature, with the increasing pressure, the interface tension decreases. At the condition of temperature *T* = 401 K (the condition of average temperature during the natural gas charging process), when pressure increase from 0.1 MPa (the atmospheric pressure) to 23 MPa (the present reservoir pressure), the interface tension decreases from 57.54 to 50.42 mN/m which decreases about 12.37%. When pressure increase to 65 MPa (the formation pressure under the natural gas charging process), the interface tension decreases to 43.82 mN/m (about 23.84% compared to the interface tension under atmospheric condition).

Figure 9b shows the variation of CH_4_/water interface tension under different temperatures under conditions of identical temperature *P* = 23 MPa (the present reservoir condition). With the increasing temperature, the interfacial tension tends to decrease. When temperature increase from 293 K (the standard temperature) to 343 K (the present reservoir condition), the interface tension decreases from 56.81 to 53.20 mN/m (6.35%), when temperature increase to 401 K (the average temperature during the natural gas charging process), the interfaces tension decreases to 50.02 mN/m (decrease 11.95% compared to the standard condition). The CH4/water interface tension decreases both with the increase temperature and pressure. It is noted that, at temperatures of 273–293 K and a pressure of 23 MPa, a drop of water can form methane hydrate^37^. However, in this work, we concentrate our research under geological condition underground. The geological formation of tight gas is typically high pressure and high temperature (More than 401 K). The gas hydrates cannot form under such geological condition in this work^38^.

## Numerical simulation set up

**Figure 10:**
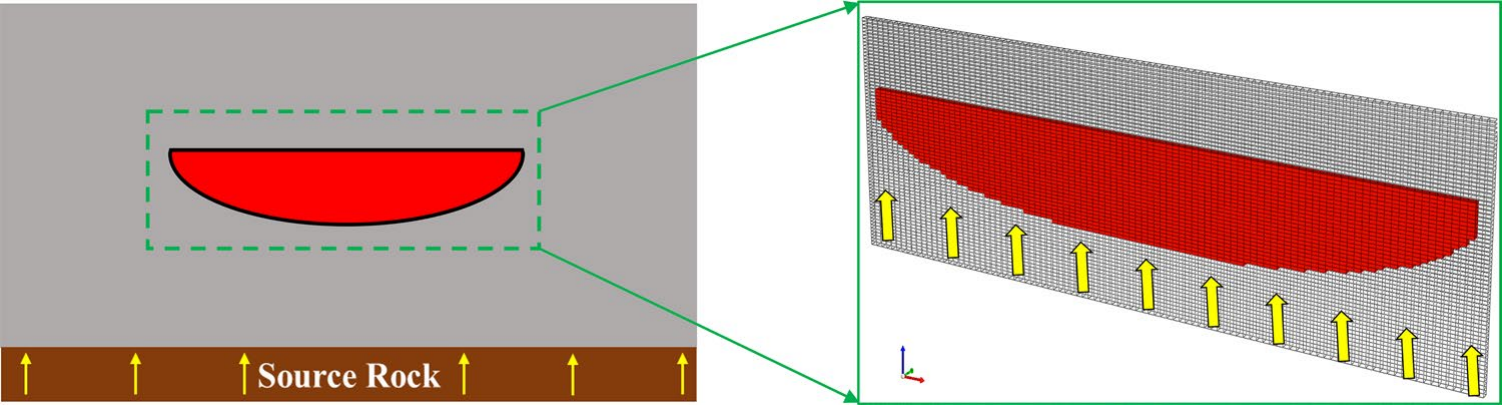
The established single sand body geological model. The red represents the sand body. The single sand body has a flat top and convex bottom, it is consistent with the profile of the river. The yellow represents natural gas. The hydrocarbon generated by the source rock is charged into the sand body through the hydrocarbon carrier system.

In this section, we establish a single sand body geological model (as shown in Fig. 10) with a convenient algorithm^3^, and the semi-analytical calculation method of contact angle and interfacial tension proposed in “Interactions in natural gas–water–rock system” is embedded into the numerical simulation program. The gas charging and accumulation process will be simulated. According the previous study and simulation method in tight gas field^3^, the composition of naturals gas considered in this work is methane (CH_4_) only, which allows us to more accurately analyze the changes in natural gas properties.

A critical requirement for gas charging is that the gas phase pressure is high enough to displace water from the formation. The threshold capillary pressure (*P*_*gE*_) for the gas phase is given as:

7 $${P}_{gE}={P}_{g}-{P}_{w}=\frac{2\sigma cos\theta }{r},$$

where *P*_*w*_ is the water pressure, *r* is the radius of the pore throats, *σ* is the water/methane interface tension and *θ* is the contact angle of the methane/water/rock system. In Eq. (7) the interface tension (IFT) *σ* and contact angle *θ* are two key parameters to determine the threshold capillary pressure.

In the reservoir numerical simulation method, the capillary force is often expressed by the mercury injection curve which can be tested by the experimental method. In this work, to reproduce the conditions in the actual situations, physical parameters from experimental testing including capillary pressure and relative permeability curves used in the simulation process are tested by real physical experiment as shown in Table 1.

**Table 1:** Parameters used in the numerical simulations.

	Porosity (%)	Permeability (× 10^3^μm^2^)	Capillary force curve (CFC)	Relative permeability curve (RPC)
S1	10.19	0.6650	CFC-1-1	RPC-1
S2	7.59	0.3350	CFC-2-1	RPC-2
S3	5.79	0.2280	CFC-3-1	RPC-3
S4	5.33	0.13	CFC-4-1	RPC-4

**Figure 11:**
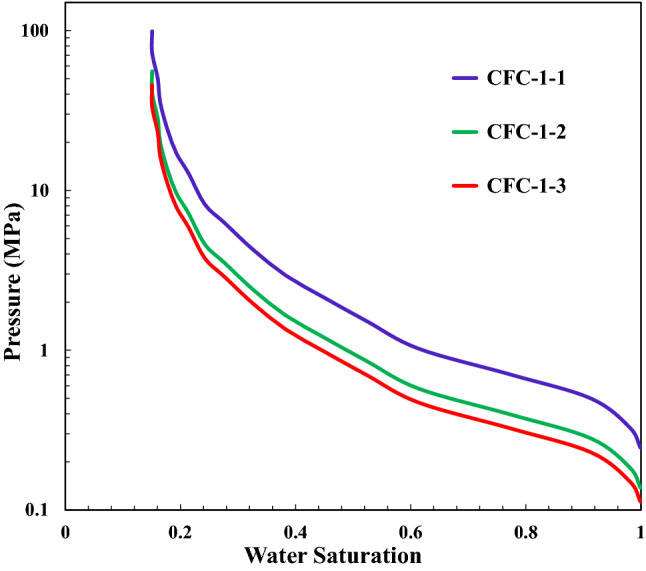
The capillary force curves under different pressure and temperature. The capillary force curve under the standard condition is obtained by experiment, the capillary force curves under present reservoir condition (CFC-1-1) and fluid charging condition (CFC-1-2 and CFC-1-3) are obtained by the method mentioned above.

The CFC-1-1, CFC-2-1, CFC-3-1, CFC-4-1 are capillary force curves of the present reservoir condition. However, with the increasing temperature and pressure, the interface tension and contact angle will decrease, which indicates that the capillary force will also decrease. With the method proposed in “Interactions in natural gas–water–rock system”, based on the present reservoir condition, under the average pressure and temperature condition when fluid charging into the formation, the capillary force will decrease 23%, and under the highest pressure and temperature condition, the capillary fore will decrease 33%. The capillary force curves for the different conditions can be calculated directly and conveniently by the proposed method above. Figure 11 shows the capillary force curves for sand core S1 under different conditions. CFC-1-1 is the capillary force curve under present reservoirs condition (Temperature = 343 K), CFC-1-2 is the capillary force curve under average pressure and temperature condition when fluid charging into the formation (Temperature = 401 K), and CFC-1-3 is the capillary force curve under highest pressure and temperature condition (Temperature = 443 K).

Table 2 shows the parameters used in all nine numerical simulations.

**Table 2:** Parameters for nine numerical simulations.

	Parameters for the single sand body				Parameters for surrounding sandstones			
	Porosity (%)	Permeability (× 10^3^ μm^2^)	CFC	RPC	Porosity (%)	Permeability (× 10^3^ μm^2^)	CFC	RPC
Simulation 1	5.79	0.2280	CFC-1-1	RPC-1	1.6	0.024	CFC-4-1	RPC-4
Simulation 2	5.79	0.2280	CFC-1-2	RPC-1	1.6	0.024	CFC-4-2	RPC-4
Simulation 3	5.79	0.2280	CFC-1-3	RPC-1	1.6	0.024	CFC-4-3	RPC-4
Simulation 4	10.19	0.6650	CFC-2-1	RPC-2	1.6	0.024	CFC-4-1	RPC-4
Simulation 5	10.19	0.6650	CFC-2-2	RPC-2	1.6	0.024	CFC-4-2	RPC-4
Simulation 6	10.19	0.6650	CFC-2-3	RPC-2	1.6	0.024	CFC-4-3	RPC-4
Simulation 7	7.59	0.3350	CFC-3-1	RPC-3	1.6	0.024	CFC-4-1	RPC-4
Simulation 8	7.59	0.3350	CFC-3-2	RPC-3	1.6	0.024	CFC-4-2	RPC-4
Simulation 9	7.59	0.3350	CFC-3-3	RPC-3	1.6	0.024	CFC-4-3	RPC-4

## Results and discussion

The results of nine simulations are shown in Table 3. Simulation 1, 4, and 7 are under present reservoir temperature and pressure conditions. Simulation 2, 5, and 8 are under average temperature and pressure conditions during the hydrocarbon migration and accumulation process. Simulation 3, 6, and 9 are under the highest temperature and pressure conditions during the hydrocarbon migration and accumulation process. Simulation 1, 2 and 3; 4, 5, and 6; 7, 8, and 9 used the same sandstone parameters.

**Table 3:** Numerical simulation results.

Simulation no.	Pore volume of the sand body (m^3^)	Gas saturation	Reserve (× 10^4^ m^3^)
1	3361	0.57	68.46
2	3361	0.58	70.44
3	3361	0.59	71.69
4	5858	0.40	79.76
5	5858	0.41	82.91
6	5858	0.42	84.89
7	4392	0.42	72.88
8	4392	0.43	76.61
9	4392	0.45	78.22

**Figure 12:**
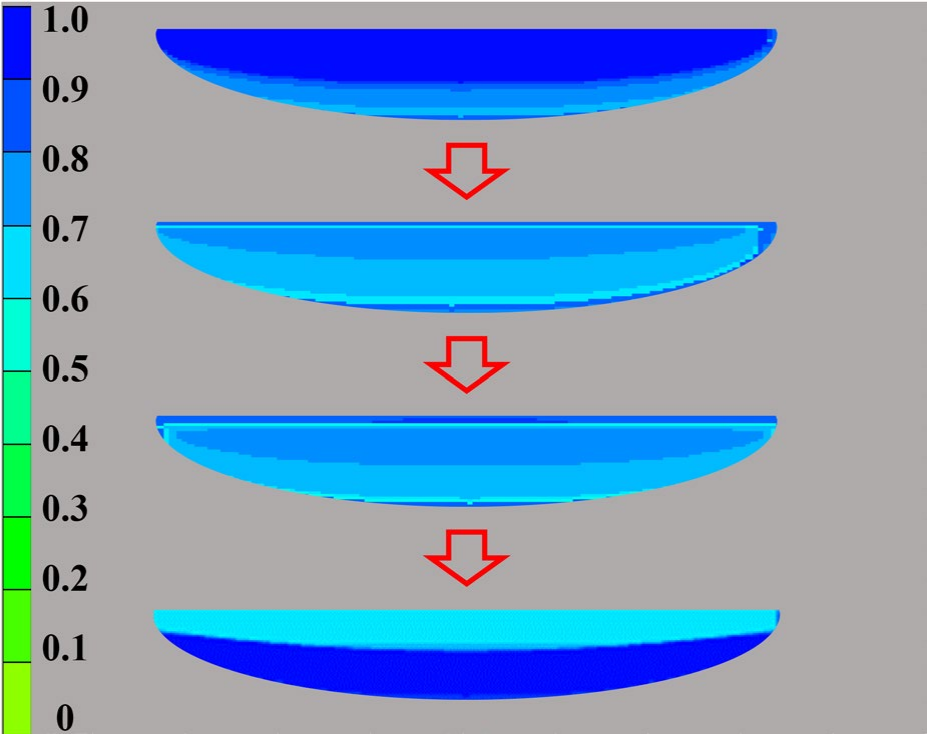
The snapshots during the gas migration and accumulation process in the sand body. The blue represents the higher water saturation, and the green represents the higher gas saturation. When the natural gas migrates into the sand body from the bottom edge, the water mainly distributes in the upper part of the sand body. After a long geological history, the water mainly distributes at the bottom of the sand body.

The simulation results are visualized and quantified. Figure 12 shows the snapshots during the simulation process. The blue represents the higher water saturation, and the green represents the higher gas saturation. When the natural gas migrates into the sand body from the bottom edge, the water mainly distributes in the upper part of the sand body. After a long geological time, the water mainly distributes at the bottom of the sand body, which is consistent of the real geological phenomenon^3^. Table 4 shows the data analysis results of nine simulations. As shown in Table 4, with the increase of temperature and pressure during the hydrocarbon migration and accumulation process, more gas will accumulate in the sand body, the reserve also increases. Compared with the natural gas reserves under the present reservoir condition, the gas reserves increase 4.27%, 6.03%, and 7.33%, respectively.

**Table 4:** Data analysis results.

Group	Simulation no.	Reserve (× 10^4^ m^3^)	Variation (%)
A	1	68.46	0.00
	2	70.44	2.89
	3	71.69	4.72
B	4	79.76	0.00
	5	82.91	3.95
	6	84.89	6.43
C	7	72.88	0.00
	8	76.61	5.12
	9	78.22	7.33

**Figure 13:**
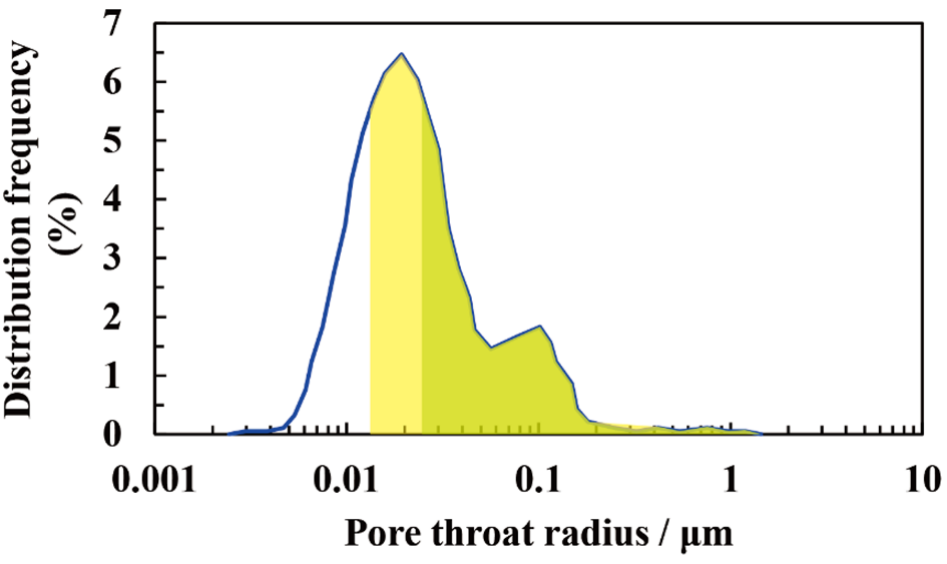
The distribution of pore throat of a tight sand core. The X-axis is the logarithmic distribution of the pore-throat size. The Y-axis is the distribution frequency of pore-throat distribution. The blue line represents the pore-throat distribution. The green represents the range of pore-throats that can be filled at current formation temperature and pressure conditions. The yellow represents the range of pore-throats that can be filled at the highest temperature and pressure during the accumulation process.

Figure 13 shows the distribution of the pore-throat of a tight sand core. The green represents the range of pore-throats that can be filled at current formation temperature and pressure conditions. The yellow represents the range of pore-throats that can be filled at the highest temperature and pressure during the accumulation process. Capillary force is the main resistance during filling, and the smaller the pore or throat is, the greater the capillary force. The pore-throat can be filled only if the charging force is greater than the capillary resistance. Under higher temperature and pressure conditions, the interface tension will decrease and the contact angle will increase, according to Eq. (7), the capillary force of the pore and throat will decrease, which indicates that more pores and throat will be filled with natural gas. This is the mechanism of why the reserve will increase with the increase of contact angle and the decrease of interface tension.

## Summary and conclusions

In this study, we simulate the natural gas migration and accumulation process considering the temperature- and pressure-dependent properties in the gas–water–rock system, and the results are visualized and quantified. The single sand body geological model is established with a convenient generating algorithm. A novel semi-analytical method based on the contact angle test experiment in real sand core from the Xujiahe Formation is proposed to calculate the temperature- and pressure-dependent contact angle and interface tension which reflects the interactions between fluid–rock and fluid–fluid. This semi-analytical is embedded in the numerical simulation during the simulation of the natural gas charging process. The hydrocarbon accumulation stage and process are also revealed by inclusions. The main conclusions are summarized as follows:

1. According to the proposed semi-analytical calculation method, with the increase of temperature and pressure, the contact angle will increase and the interface tension between natural gas and water will decrease. The capillary resistance in the formation will be reduced.
2. With the decrease of capillary resistance, the natural gas can be charged into smaller pores, so that the actual charging threshold is lower than the one originally obtained under present reservoir conditions. After considering the temperature and pressure during the accumulation process, some sand bodies that were thought not to be charged may be charged.
3. According to the fluorescence spectroscopy, natural gas tends to migrate and accumulate in the oil-wet (the contact angle is larger) formation. The well in these areas has larger production. This is consistent with our simulation results.

## Supplementary Information

Supplementary Information.
