# Supplementary material for: Effects of interactions in natural gas/water/rock system on hydrocarbon migration and accumulation
Source: Sci Rep. 2021 Nov 11;11:22070. doi: 10.1038/s41598-021-01653-0 (PMC8586332; doi:10.1038/s41598-021-01653-0)
Supplement: Supplementary file 1 — Supplementary Information. [file 41598_2021_1653_MOESM1_ESM.pdf]

Appendix

Table A1. The collected data used to reproduce Fig. 1

Researchers	Method	T(K)	P (Mpa)	IFT (mN/m)	△ρ (g/cm3)	T/Tr (dimensionless)	Calculated IFT(mN/m)	Error (%)
Jennings et al. (1971)	Experiment	296.45	0.10	72.00	0.99	1.56	70.26	-2.41
			10.10	64.20	0.94	1.56	61.99	-3.44
			20.10	57.80	0.91	1.56	58.09	0.50
			25.10	56.20	0.89	1.56	55.86	-0.60
			30.00	53.90	0.88	1.56	54.85	1.76
			35.00	52.80	0.87	1.56	53.90	2.08
			40.00	51.70	0.86	1.56	53.01	2.53
Sachs et al.(1995)	Experiment	298.15	10.39	60.96	0.92	1.56	59.36	-2.63
			10.39	60.91	0.92	1.56	59.36	-2.55
			10.40	60.87	0.92	1.56	59.36	-2.49
			10.40	60.73	0.92	1.56	59.36	-2.26
			10.40	60.80	0.92	1.56	59.36	-2.38
			21.25	55.50	0.84	1.56	51.25	-7.66
			21.25	55.65	0.84	1.56	51.24	-7.93
			35.49	52.90	0.77	1.56	50.56	-4.42
			36.52	52.63	0.77	1.56	50.42	-4.20
			36.53	52.90	0.77	1.56	50.42	-4.69
			36.53	52.71	0.77	1.56	50.42	-4.35
			46.85	51.83	0.75	1.56	49.36	-4.77
			46.88	52.06	0.75	1.56	49.35	-5.20
Sachs et al. (1996)	Experiment	298.15	0.10	72.20	0.99	1.56	70.26	-2.68
			10.20	60.70	0.94	1.56	61.99	2.13
			20.10	55.70	0.91	1.56	58.09	4.29
			29.90	54.00	0.88	1.56	54.85	1.57
			40.00	52.50	0.86	1.56	53.01	0.97
		373.15	0.10	59.00	0.98	1.96	57.74	-2.13
			10.20	52.50	0.93	1.96	53.07	1.10
			20.10	48.50	0.90	1.96	50.88	4.90
			30.00	46.90	0.87	1.96	49.06	4.60
			40.00	46.10	0.84	1.96	47.56	3.17
Lepski et al. (1997)	Experiment	325.70	10.00	62.60	0.94	1.71	58.23	-6.98
			13.80	59.30	0.93	1.71	57.11	-3.70
			17.10	56.70	0.91	1.71	55.06	-2.90
			20.70	57.90	0.90	1.71	54.12	-6.52
			20.70	55.30	0.90	1.71	54.12	-2.13
			24.10	53.70	0.89	1.71	53.25	-0.85
		351.60	10.30	58.90	0.94	1.84	55.74	-5.36
			13.80	56.60	0.92	1.84	53.89	-4.78
			17.30	54.70	0.91	1.84	53.05	-3.02
			24.10	53.90	0.89	1.84	51.51	-4.43
		368.60	10.30	55.30	0.93	1.93	53.47	-3.31

			13.80	55.10	0.92	1.93	52.67	-4.42
			17.20	52.50	0.91	1.93	51.91	-1.13
			20.60	52.10	0.90	1.93	51.20	-1.73
			24.10	51.10	0.88	1.93	49.90	-2.34
Tian et al. (1997)	Experiment	373.00	10.00	52.40	0.94	1.96	53.90	2.86
			20.00	50.20	0.90	1.96	50.88	1.35
			40.00	46.30	0.84	1.96	47.56	2.72
			60.00	45.40	0.79	1.96	45.65	0.55
			100.00	43.90	0.73	1.96	44.10	0.45
Ren et al. (2000)	Experiment	298.15	1.00	72.00	0.99	1.56	70.26	-2.41
			5.10	66.90	0.97	1.56	66.66	-0.36
			10.10	61.40	0.94	1.56	61.99	0.96
			15.10	58.70	0.92	1.56	59.31	1.04
			20.00	56.00	0.91	1.56	58.09	3.73
			30.00	54.00	0.88	1.56	54.85	1.57
		373.15	1.00	59.00	0.97	1.96	56.70	-3.90
			5.05	56.10	0.96	1.96	55.71	-0.70
			10.20	53.20	0.93	1.96	53.07	-0.24
			15.10	50.90	0.92	1.96	52.30	2.75
			20.10	48.70	0.90	1.96	50.88	4.47
			30.00	47.00	0.87	1.96	49.06	4.38
Rivera et al. (2003)	MD	150.00	20.00	33.09	0.72	1.96	33.49	1.21
		200.00	20.00	25.59	0.68	1.96	24.59	-3.91
		270.00	20.00	19.19	0.60	1.96	20.19	5.21
		336.00	20.00	12.37	0.47	1.96	11.44	-7.52
Biscay et al. (2009)	MD(TA)	373.15	1.00	57.40	0.97	1.96	56.70	-1.23
			5.12	53.90	0.96	1.96	55.71	3.36
			10.10	50.00	0.94	1.96	53.90	7.80
			15.00	48.10	0.92	1.96	52.30	8.73
			20.00	46.60	0.90	1.96	50.88	9.18
			30.20	45.90	0.87	1.96	49.06	6.88
			40.00	45.00	0.84	1.96	47.56	5.69
			50.00	44.10	0.81	1.96	46.34	5.07
	MD(IK)	373.15	1.00	58.40	0.97	1.96	56.70	-2.92
			5.12	55.40	0.96	1.96	55.71	0.56
			10.10	51.10	0.94	1.96	53.90	5.48
			15.20	49.30	0.92	1.96	52.30	6.08
			20.00	48.00	0.90	1.96	50.88	6.00
			30.10	47.30	0.87	1.96	49.06	3.72
			50.00	45.80	0.81	1.96	46.34	1.17
	MD(KBZ)	373.15	1.00	57.40	0.97	1.96	56.70	-1.23
			5.12	55.10	0.96	1.96	55.71	1.11
			10.20	51.30	0.93	1.96	53.07	3.46
			15.20	49.20	0.92	1.96	52.30	6.30
			20.00	47.90	0.90	1.96	50.88	6.22

			30.40	47.50	0.86	1.96	48.53	2.16
			50.00	45.40	0.81	1.96	46.34	2.06
	MD(KB)	373.15	50.00	47.90	0.81	1.96	46.34	-3.27
			49.90	45.00	0.81	1.96	46.34	2.97
			40.20	45.90	0.84	1.96	47.56	3.62
			29.90	46.90	0.87	1.96	49.06	4.60
			19.80	47.40	0.90	1.96	50.88	7.34
			14.90	49.10	0.92	1.96	52.30	6.51
			10.20	52.50	0.93	1.96	53.07	1.10
			4.96	56.50	0.96	1.96	55.71	-1.40
			1.00	58.20	0.97	1.46	70.65	21.39
Shariat et al.(2014)	Experiment	323.15	7.85	69.35	0.95	1.70	59.66	-13.98
			15.36	63.65	0.92	1.70	56.24	-11.64
			20.82	60.43	0.90	1.70	54.29	-10.17
		373.15	7.51	60.93	0.95	1.96	54.78	-10.10
			13.65	56.97	0.92	1.96	52.30	-8.20
			21.50	53.75	0.89	1.96	50.23	-6.54
Kashefi et al. (2016)	MD	311.00	3.40	66.03	0.97	1.63	64.33	-2.58
			7.54	59.95	0.95	1.63	61.42	2.46
			14.38	57.94	0.92	1.63	57.67	-0.47
			28.21	52.29	0.88	1.63	53.63	2.55
			44.73	51.43	0.85	1.63	51.20	-0.44
			61.30	49.28	0.82	1.63	49.22	-0.13
			81.60	50.39	0.80	1.63	48.10	-4.54
		373.00	3.65	54.76	0.96	1.96	55.71	1.73
			10.07	50.64	0.94	1.96	53.90	6.44
			21.14	48.29	0.89	1.96	50.23	4.02
			30.54	45.02	0.86	1.96	48.53	7.79
			51.02	42.20	0.81	1.96	46.34	9.80
			60.31	41.61	0.79	1.96	45.65	9.71
			71.05	41.92	0.77	1.96	45.05	7.48
			82.76	41.93	0.75	1.96	44.54	6.23
Yang et al. (2017)	MD	311.00	2.53	62.80	0.98	1.63	65.91	4.96
			2.53	60.90	0.98	1.63	65.91	8.23
			5.38	58.20	0.96	1.63	62.83	7.96
			14.20	54.90	0.93	1.63	58.85	7.19
			21.50	53.50	0.90	1.63	55.52	3.78
			36.40	52.90	0.86	1.63	51.96	-1.78
			51.60	52.10	0.83	1.63	49.84	-4.35
			68.70	51.90	0.81	1.63	48.64	-6.28
			83.90	52.10	0.80	1.63	48.10	-7.67
			89.90	51.90	0.80	1.63	48.10	-7.31
		373.00	2.85	52.48	0.97	1.96	56.70	8.03
			5.70	50.42	0.95	1.96	54.78	8.64
			25.00	46.75	0.88	1.96	49.63	6.15
			38.92	46.57	0.84	1.96	47.56	2.13

			56.33	45.99	0.80	1.96	45.98	-0.02
			67.72	46.43	0.78	1.96	45.34	-2.35
			81.65	46.46	0.75	1.96	44.54	-4.13
			90.51	46.27	0.74	1.96	44.31	-4.23
Li et al., (2019)	MD	278.15	1.00	66.20	0.99	1.46	74.83	13.04
			3.21	64.64	0.98	1.46	72.68	12.44
			4.82	63.97	0.97	1.46	70.65	10.44
			6.72	62.85	0.96	1.46	68.74	9.36
			8.03	61.73	0.95	1.46	66.93	8.42
			9.64	60.61	0.95	1.46	66.93	10.43
			10.95	60.39	0.94	1.46	65.23	8.01
			12.41	59.16	0.94	1.46	65.23	10.26
			15.47	57.60	0.93	1.46	63.63	10.46
			19.56	57.15	0.91	1.46	60.70	6.21
			25.11	55.70	0.90	1.46	59.36	6.58
			32.41	55.14	0.88	1.46	56.94	3.26
			42.63	54.58	0.87	1.46	55.83	2.30
			57.52	54.58	0.85	1.46	53.83	-1.37
			80.00	53.58	0.83	1.46	52.08	-2.80
		298.15	1.00	68.33	0.99	1.56	70.26	2.83
			2.92	66.99	0.98	1.56	68.41	2.12
			4.67	64.75	0.97	1.56	66.66	2.95
			6.13	63.97	0.96	1.56	65.01	1.63
			7.30	63.08	0.96	1.56	65.01	3.06
			8.47	61.96	0.95	1.56	63.46	2.42
			9.64	60.73	0.95	1.56	63.46	4.49
			13.58	58.94	0.93	1.56	60.61	2.83
			16.93	57.04	0.92	1.56	59.31	3.98
			21.02	57.04	0.90	1.56	56.94	-0.18
			27.01	55.37	0.89	1.56	55.86	0.89
			35.33	55.26	0.87	1.56	53.90	-2.46
			48.61	54.47	0.85	1.56	52.17	-4.22
			68.18	54.92	0.82	1.56	49.98	-8.99
			80.00	55.25	0.81	1.56	49.35	-10.69

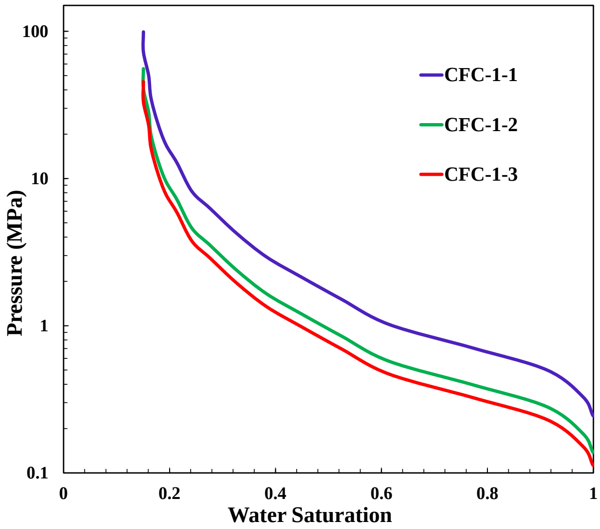

CFC-1

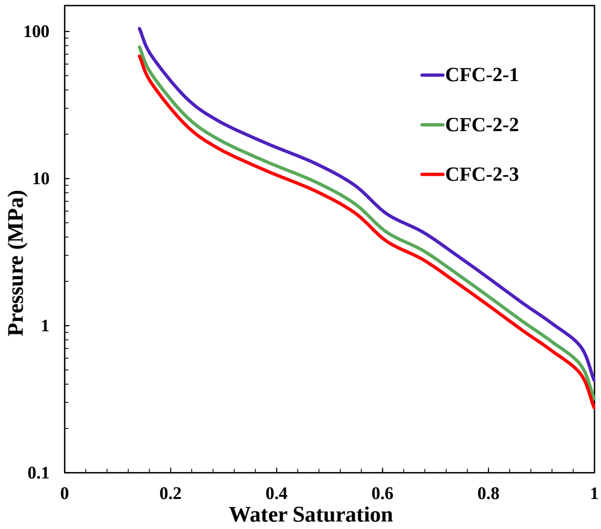

CFC-2

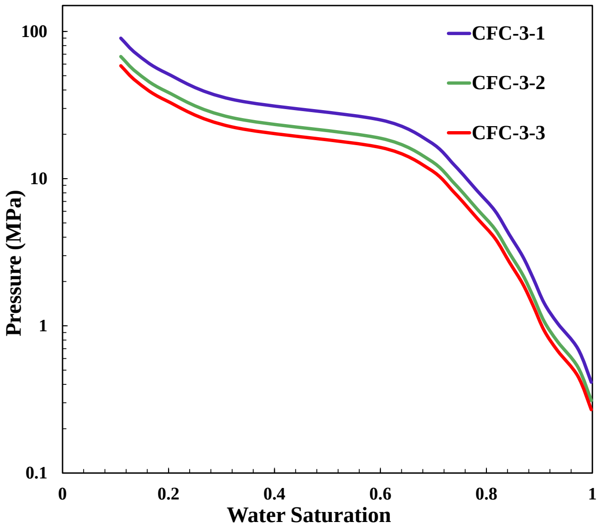

CFC-3

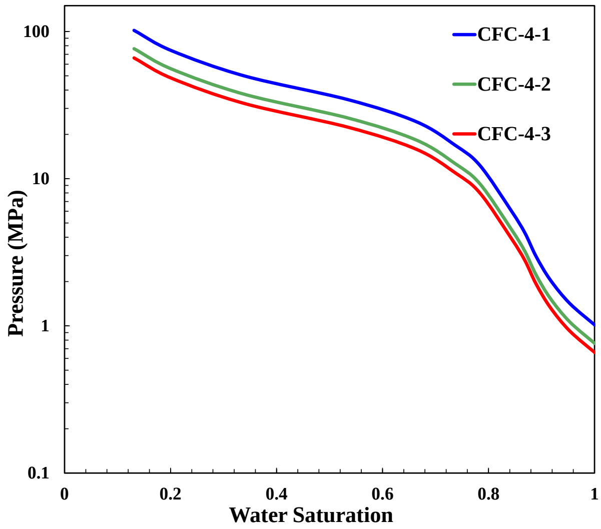

CFC-4

Fig. A1. The capillary force curves used in the numerical simulation.
